# Regional autozygosity association with albumin-to-creatinine ratio reveals a novel *FTO* region in an Indigenous Australian population

**DOI:** 10.1038/s41431-025-01799-9

**Published:** 2025-02-24

**Authors:** Vignesh Arunachalam, Kim N. Tran, Wendy Hoy, Rodney A. Lea, Shivashankar H. Nagaraj

**Affiliations:** 1 Centre for Genomics and Personalised Health, School of Biomedical Sciences, Queensland University of Technology, Brisbane, QLD Australia; 2 Faculty of Medicine, University of Queensland, Brisbane, QLD Australia

**Keywords:** Genome-wide association studies, Chronic kidney disease

## Abstract

The genetic distinctiveness of Indigenous Australian populations is well established, yet the Tiwi population remains underrepresented in genetic research. Due to their prolonged geographic isolation, these populations are prone to increased runs of homozygosity (ROH). We investigated the genetic diversity of the Tiwi population, isolated from mainland Australia for decades, based on ROH and their associations with clinical traits. We analyzed 455 whole genome sequences to identify population structure via PCA and performed a comparison with UK Biobank, Melanesian, and Polynesian cohorts. ROH assessment and genome-wide and regional measures of homozygosity were used to explore associations between clinical traits and autozygosity. Our analysis revealed distinct genetic characteristics of the Tiwi population that aligned closely with those of the Melanesian cohort. Tiwi individuals exhibited an increased burden of ROH, particularly in *LINC0109*, *FMLN1*, and *RPL17P45* genes on chromosomes 2, 17, and 18, respectively, indicating prolonged isolation and genetic drift. A positive correlation was observed between genomic F_*ROH*_ and albumin-to-creatinine ratio (ACR) levels, suggesting a potential link between autozygosity and renal health markers. Furthermore, regional autozygosity association analysis revealed an association between elevated ACR and a region in *FTO*, implicating its role in obesity, kidney disease, and cardiovascular conditions. Importantly, we found that this association is strong under the recessive model. This research lays a robust foundation for further exploration of ROH mapping and its implications for disease susceptibility within Indigenous communities worldwide.

## Background

The genetic history of Aboriginal Australians reveals a single founding population ~10–32 thousand years ago, suggesting a potential selection signal related to desert habitation [1]. Indigenous people, who make up 3.8% of the Australian population (*n* = 984,000) [2], exhibit high genetic distinctiveness and genetic variation [3–5]. Despite the global advancement in genomics, its application to the Indigenous population remains underexplored [6]. This is partly due to the lack of Indigenous-specific data in major databases, such as 1000 Genomes [7], the Genome Aggregation Database (gnomAD) [8], and the Human Genome Diversity Project (HGDP) [9]. Up to 34% of Indigenous-specific single nucleotide variants (SNVs) are missing from these datasets [10], and only 0.05% of Indigenous Australians are represented in genomic studies, highlighting a significant representation gap [11]. This underscores the need for more comprehensive Indigenous genomic databases to advance genomic medicine and ensure equitable healthcare [12].

Formerly known as the Sentinel Islands because of their strategic location 60 km north of Darwin in the Timor Sea, the Tiwi Islands are Australia’s largest islands after Tasmania, comprising Melville and Bathurst Islands, with a collective population of 2348 [13]. The Tiwi people lived in prolonged isolation from mainland Australia, preserving a distinct language and culture divergent from other Aboriginal Australian communities. Genomic studies reveal significant genetic differentiation in the Tiwi population due to their isolation from rising sea levels, characterized by unique genetic drift patterns, prolonged isolation, and minimal migration [10]. Our recent research highlighted a higher prevalence of kidney disease and greater heritability for urinary albumin-to-creatinine ratio (ACR) in the Tiwi and a unique kidney genetic profile compared to global populations [14].

We hypothesized that the Tiwi population exhibits a higher degree of autozygosity (F_*ROH*_), as measured from runs of homozygosity (ROH). ROH are uninterrupted, identical DNA sequences arising when an individual inherits two identical haploid copies of a sequence from both parents. Smaller isolated populations typically have more ROH compared to admixed populations [15, 16]. Therefore, ROH is a valuable tool for exploring population structures and disease genetics, with insights into the history and genetic architecture of complex diseases [17]. ROH are associated with the risk of schizophrenia [18], Alzheimer’s disease [19], autism [20], coronary artery disease [21], and cognitive impairment [22].

Despite these reports, ROH and their association with disease traits have not been studied in the Tiwi population. However, recent studies on Tiwi genomes demonstrated over 10% extended homozygosity, exceeding that of Indigenous American populations threefold and Eurasian populations tenfold, likely attributable to increased background relatedness rather than consanguinity commonly observed in population isolates [10]. Notably, a previous Tiwi study speculated that there might be a potential link between kidney disease and shorter ROH. However, this study found no association between the longer ROH and ACR levels, which could be attributed to high genotyping error or low coverage rates [5]. Given the unique genetic makeup and historical context of the Tiwi population, investigating ROH patterns and their association with clinical traits is essential for understanding their relevance to population health and disease susceptibility. Previous studies conducted on the Tiwi population have not fully captured the breadth of ROH regions and their association with kidney traits, despite the high prevalence of chronic kidney conditions in this population [14, 23]. This study aims to investigate ROH prevalence, extent, and their relationship with kidney markers, establishing a foundation for ROH mapping to better understand disease susceptibility within the Tiwi community.

## Methods

### Study population and datasets

The whole genome sequencing (WGS) data for the Tiwi population were obtained from the blood samples of 497 Tiwi individuals collected between 2013 and 2014, representing approximately 40% of Tiwi adults [14]. This project conducted in consultation with Tiwi Elders and Indigenous research experts, adheres to NHMRC guidelines and core values of Spirit and Integrity, Cultural Continuity, Equity, Reciprocity, Respect, and Responsibility, with participants’ consent obtained for using blood samples to investigate chronic kidney disease (CKD) [5, 14] The associated phenotypic profiles include blood pressure, height, weight, waist circumference, glycated hemoglobin, serum and urine albumin, serum and urine creatinine, urinary ACR, and estimated Glomerular Filtration Rate (eGFR) (estimated using CKD-EPI 2021 equation [24]). The quality control (QC) steps were performed using Plink v1.9 which included missingness rate (--geno 0.02 –mind 0.1), Hardy-Weinberg equilibrium (*p* > 5*10^−8^), heterozygosity rate (±3 standard deviation), and minor allele frequency (--maf 0.05) [14]. Following QC, we utilized 455 samples and 4.9 million SNPs for further downstream analysis.

A total of 150,000 WGS and corresponding phenotype data were obtained from the UK Biobank (UKBB) database for comparative analyses [25]. The UKBB populations were analyzed separately according to their self-reported ethnicity which included African (*n* = 1320), British (*n* = 124,948), Caribbean (*n* = 1835), Chinese (*n* = 415), Indian (*n* = 1772), Irish (*n* = 3779), and Pakistani (*n* = 654) populations. We also obtained the genotyping array data for the Pacific Islands population including Polynesian and Melanesian (*n* = 165) [26], which captures common variants across 25 different populations; however, data from only Polynesian and Melanesian ethnicities were used for ROH analysis. The same QC steps used for the Tiwi population were applied to the UKBB to ensure consistency. We also downloaded ROH summary data from Joshi et al., which includes populations such as Amish, Hutterites (HUTT), African, European isolate, Norfolk Isolate, and Hispanic [22].

### Population structure

To compare the population structures, we performed a principal component analysis (PCA) using autosomal SNPs that met QC criteria. Initially, we generated a PCA plot for the Tiwi and UKBB populations. We filtered and merged shared common variants (AF > 5%) to create a unified Plink file, followed by LD pruning with the Plink command *–indep-pairwise 50 5 0.2*. This step was repeated for the Polynesian and Melanesian populations alongside the Tiwi population. Due to the limited number of independent variants shared across all groups, we did not combine all populations in a single plot. Instead, we analyzed the Tiwi and UKBB populations separately from the isolated and the Tiwi populations. PCA was performed using Plink’s *–pca* function, and the resulting principal components (PCs) were visualized using the *ggplot2* [27] package in Rv4.0.3.

### ROH identification

ROH identification was performed on the common variants using the default parameters of the *–homozyg* option in PLINK v1.9, which calls ROH using a sliding window that scans along an individual’s SNP data to detect homozygous stretches [15, 22]. Uniform parameters were applied to detect ROH across numerous populations, including Tiwi, British, Caribbean, Chinese, Indian, Irish, Pakistani, Melanesian, and Polynesian. These datasets were used to compare the extent of ROH in the Tiwi population to other global and isolated populations.

The following measures of homozygosity were calculated in this study: the average number of ROH, the average total length of ROH, and the proportion of the autosomal genome covered by ROH (F_*ROH*_). The average number of ROH is the total number of runs divided by the number of samples. The average total length of ROH is defined as the total ROH length divided by the number of samples. We used these two measures to compare ROH between the Tiwi and other populations. F_*ROH*_ is a measure of individual autozygosity, the ratio of the total length of ROH to the length of autosomal genomes (3 × 10^9^) covered by SNPs. Additionally, we used Spearman’s correlation to identify the association between F_*ROH*_ and various traits, with non-parametric correlation accounting for the non-normality distribution of the traits. Multiple test correction was applied using the Benjamini-Hochberg (BH) method.

### Regional ROH association

The Plink ROH results provided information about the ROH regions identified in all the individuals. For each sample, we extracted the ROH inclusion status for each SNP, assigning a value of 1, if the SNP was within the ROH region and 0, if not, representing regional autozygosity. We used a linear mixed model to explore the association between autozygosity estimates and traits, with phenotype as the outcome variable and autozygosity status as the independent variable [15]. This model included the top two genotype PCs, gender, and age as fixed covariates. Additionally, a genomic relationship matrix was included as a random effect to account for relationships among the individuals. We excluded samples lacking ROH regions and SNPs with a regional autozygosity frequency below 1%. The statistical significance threshold was determined using Bonferroni correction for the number of SNPs tested (*n* = 410k), resulting in a threshold of *p* = 1.21 × 10^−8^. Furthermore, we used Plink’s *--clump* function to identify independent signals by grouping correlated SNPs based on Linkage Disequilibrium (LD). We validated the genotype’s significant ROH variants and their association with ACR levels using additive and recessive models, adjusting for covariates including age, sex, and the top two genotype PCs, and performed using PLINK 2.0 [28].

## Results

### Tiwi population structure

**Fig. 1:**
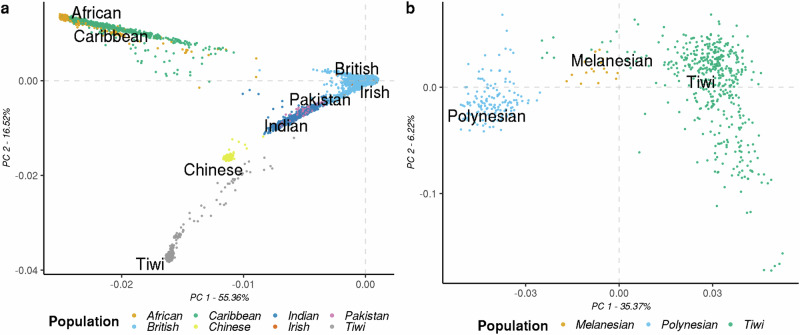
Population Structure of the Tiwi population. **a** PCA plot of Tiwi and the UK Biobank populations (self-reported ethnicities), including African, Caribbean, Indian, Pakistani, British, Chinese, and Irish populations. PC1 and PC2 explained about 72% of the variance in the data, which consisted of 114,957 independent common SNPs among the different populations. **b** PCA plot of Tiwi and isolated populations, including Polynesian, and Melanesian populations. PC1 and PC2 explained about 42% of the variance in the data, which consisted of 1863 independent common SNPs among the populations.

The WGS data from the Tiwi population identified 19,339,732 SNVs, with 42.35% of these variants absent in the UKBB datasets. In the first PCA using the Tiwi and UKBB cohorts, PC1 and PC2 explained about 71.9% of the variance. The Tiwi cohort formed a distinct cluster within the UKBB dataset (Fig. 1a). Moreover, the Tiwi population exhibited closer associations with Chinese ethnicities compared with African, Caribbean, British, and Irish populations. In the second PCA comparing Tiwi and other isolated populations, including those of Polynesian and Melanesian from the Pacific Islands, PC1 and PC2 explained about 41.6% of the variance. Notably the Tiwi cohort remained distinct (Fig. 1b). As expected, the Tiwi Islanders clustered closer to the Melanesian population than the Polynesians. Our study utilizing UKBB data and isolated populations corroborates findings from previous investigations on the Tiwi cohort [3–5, 10] and highlights distinctive genetic attributes in the Tiwi population.

### ROH prevalence in the Tiwi population compared with other isolated populations

**Fig. 2:**
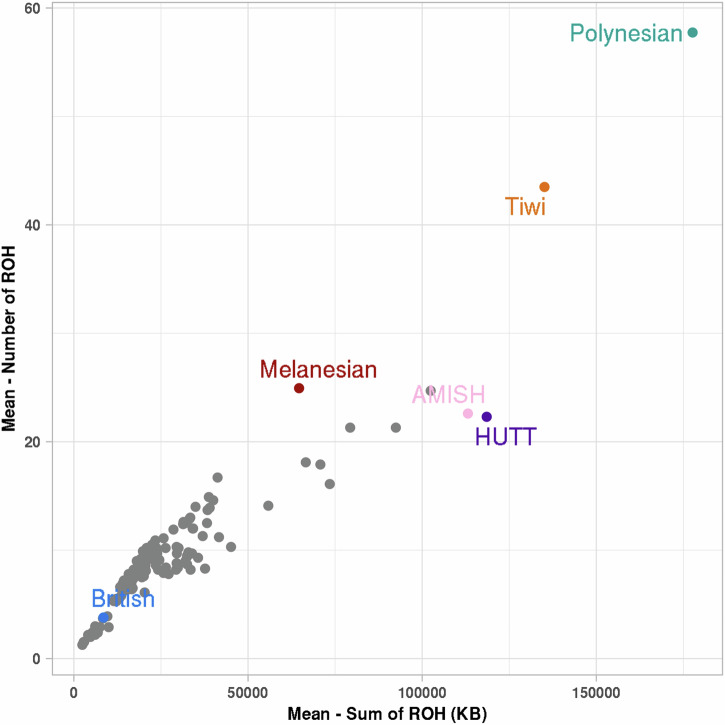
Average number of ROH and average total length of ROH in various cohorts. Tiwi individuals had a higher number of ROH compared to other ethnicities present in the UK Biobank, HUTT, and Amish populations.

The Tiwi people demonstrated a significantly higher prevalence of longer ROH than general and isolated populations (Fig. 2). For instance, the average total number of ROH was 43.5 (SD = 19.3), covering an average genomic length of 135 Mb (SD = 71 Mb) (Fig. 2 and Supplementary Table S1). In comparison, the Amish and HUTT native populations exhibited an average total number of ROH of 22.6 (SD = 6.1) and 22.3 (SD = 6.3), covering an average genomic length of 113 Mb (SD = 45 Mb) and 118 Mb (SD = 53 Mb) respectively, which were significantly lower than the Tiwi population. In contrast, the Polynesian population exhibited more ROH compared with the Tiwi population. Additionally, the Tiwi individuals showed a significantly higher prevalence of ROH compared to other isolates and all ethnicities in the UKBB population (Fig. 2).

### ROH distribution in the Tiwi population

**Fig. 3:**
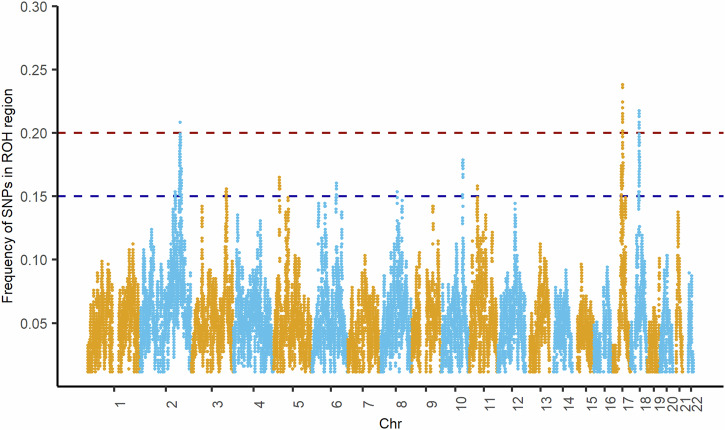
Distribution of Runs of Homozygosity (ROH) in the Tiwi Cohort. Manhattan plot showing the frequencies of SNPs present in ROH across the genome. The y-axis represents the frequency of SNPs present in the ROH region, and the x-axis represents the chromosomes. The blue dotted line represents a frequency of 0.20, whereas the red dotted line represents a frequency of 0.15.

ROH were found on every autosomal chromosome and extensively distributed across the Tiwi genomes. On average, each participant had 43.5 ROH segments >1.6 Mb, spanning a total of 135 Mb. The genome-wide SNP frequency in the ROH region is illustrated in Fig. 3, and Table 1 provides the most predominant ROH regions (≥20%). Notably, 4.0% (*n* = 18) of individuals had no ROH segments, 1.3% (*n* = 6) had shorter ROH regions (≤1.6 Mb), and the remaining 94.7% had longer ROH segments (>1.6 Mb).

**Table 1:** Enriched ROH regions in the Tiwi individuals.

Chromosome	Start	End	No. of SNPs	*N* (%)	Genes mapped
2	188129455	188285982	234	91 (20)	LINC01090
17	45214912	45254071	39	96 (21.1)	FMLN1
17	45254648	45287723	36	91 (20)	SPATA32, MAP3K14
17	45299081	45856890	21	94 (20.6)	CRHR1, SPPL2C
18	40142800	40431605	434	95 (20.8)	ENSG0000286844 (uc288ffz)

Characteristics of the most frequent ROH regions in the Tiwi population (observed in ≥20% of individuals) are presented, including the chromosome number, location (start and end), number of SNPs involved, frequency, and corresponding genes within each region.

The most prevalent ROH region was on chromosome 17, followed by chromosomes 2 and 18. On chromosome 17, a region spanning 45214912–45254071 was found in 96 of 455 individuals; including 39 common SNPs mapped to the *FMLN1* gene (Table 1 and Fig. 3). Another ROH region on chromosome 17 was detected in 20.0% of individuals, encompassing 21 common SNPs. Besides chromosome 17, notable ROH regions were identified on chromosome 18 (40142800–40431605) observed in 20.9% of individuals, containing 434 common SNPs in the region 18q12.3, mapped to *ENSG00000286844* (uc288ffz), and chromosome 2 (188129455–188285982; 20.0% of individuals), which mapped to the long intergenic non-protein coding RNA 1090 (*LINC01090*) gene associated with post-traumatic stress disorder (PTSD). ROH frequencies ranging between 0.15 and 0.20 are given in Supplementary Table S2.

### Association of genome-wide ROH with CKD-related phenotypes

We analyzed 15 phenotypes, including ACR, eGFR, serum creatinine, serum albumin, HbA1c, urine albumin, urine creatinine, basic anthropometrics, and blood pressure. The strongest correlation was with urinary ACR (r = 0.182, p.adj = 0.002), followed by serum albumin (r = −0.159, p.adj = 0.007). Interestingly, eGFR (r = −0.096, p.adj = 0.10) was not correlated with F_*ROH*_. Additionally, glycemic control (HbA1c) had a weak correlation with F_*ROH*_ (r = 0.109, p.adj = 0.066), which did not withstand the BH correction (Supplementary Table S3).

### Regional autozygosity association with ACR levels

**Fig. 4:**
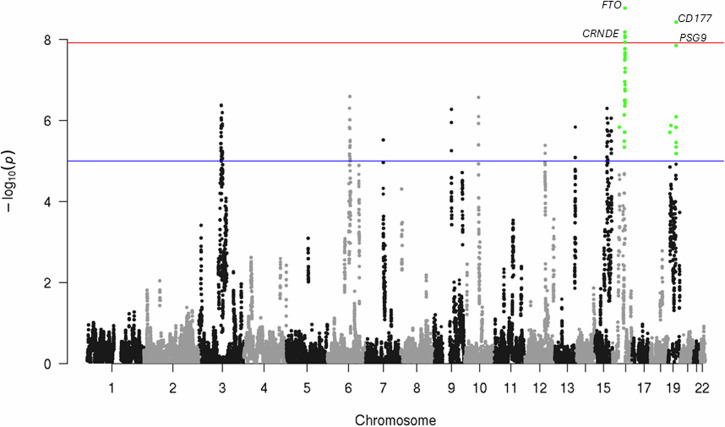
Manhattan plot of regional ROH associations with ACR genome-wide associations. The SNPs that passed the red dashed line (1.21 × 10^−8^) demonstrated genome-wide significance, whereas SNPs that passed the blue dashed line (1 × 10^−5^) demonstrated nominal significance.

We analyzed 15 phenotypes for association with regional autozygosity levels and identified one statistically significant association at the Bonferroni-corrected level (*p* = 1.21 × 10^−8^) (Fig. 4). We found that regions in chromosomes 16 and 19 were significantly associated with ACR. Increased ACR levels were significantly associated with ROH at a region of chromosome 16 that mapped to *FTO* and *CRNDE*. Additionally, increased ACR levels were significantly associated with regional autozygosity at a region of chromosome 19 that harbored members of the pregnancy-specific glycoprotein (PSG) family of genes (*PSG1, PSG4*, and *PSG9*), *CD177*, and *PRG1*.

In our validation using traditional GWAS, we could not replicate the associations identified through regional autozygosity analysis (Supplementary Table S5). However, many significant variants in these regions are notably more prevalent in the Tiwi population compared to the UKBB population. For instance, the SNPs in the gene CRNDE (chr16:54929371:G:T) and PSG1 (rs375340094) are either rare or absent in the UKKB population (Supplementary Table S4). The allele frequencies of these variants in the Tiwi population ranged from 9.9% to 68.2%, in contrast to 2.9% to 43.3% in the UKBB population, highlighting significant differences. Additionally, 1476 loci associated with ACR achieved genome-wide significance using the ROH approach, while 11 loci were detected using the traditional GWAS approach [14]. Interestingly, none of the genome-wide loci identified in regional autozygosity association reached either genome-wide or nominal significance using the traditional GWAS approach. These findings suggest that a recessive model may better explain ACR levels rather than the conventional additive approach.

### Recessive-based SNP analysis

We applied PLINK2.0 *--glm recessive* function to model ACR levels in the ROH-associated region, accounting for covariates such as age, sex, and genetic PCs. We found a strong association (lowest *p*-value = 3.19 × 10^−68^) between ACR levels and SNPs in the chromosome 16 region (Supplementary Table S6), consistent with the ROH results. However, SNPs in the chromosome 19 region (lowest *p*-value = 8.18 × 10^−7^), previously identified in the ROH analysis, showed nominal association under the recessive model. Supplementary Figure S1 illustrates the effect sizes, 95% confidence intervals, and significance levels for the top independent regional autozygosity SNPs across the additive, recessive, and regional autozygosity models. These results confirm our speculation that ACR could be associated with identified regions in the recessive model.

## Discussion

In this study, we examined the genetic distinctness of the Tiwi individuals through population structure and ROH analysis, and investigated the association between regional autozygosity and kidney markers. Our research significantly advances the understanding of the Tiwi population’s distinctive features, particularly concerning ROH and their association with kidney function, and represents the largest genetic study on the association between ROH and clinical phenotypes in this population. As a result, population structure analysis using PCA revealed distinct clustering patterns specific to the Tiwi population compared to the UKBB population. The PCA plot indicated that the Tiwi cohort clustered closely with the Chinese population, followed by Indian and Pakistani populations, and was distant from Caucasian and African populations (Fig. 1). These findings are consistent with those of previous studies on the Tiwi population [3–5]. In contrast, PCA between the Tiwi population and isolated populations including Polynesian and Melanesian cohorts revealed that the Tiwi population formed a distinct cluster; however, it was closest to the Melanesian population. This suggests the possibility of recent East Asian admixture in some Tiwi individuals, as indicated by the gradient shift toward the Chinese, Indian, and British populations in Fig. 1a, and the Melanesians in Fig. 2a. This likely reflects more recent admixture in the Tiwi population rather than deep shared ancestry. Notably, nearly half of the variants present in the Tiwi population were absent in the UKBB populations. These findings underscore the unique genetic profile of the Tiwi individuals and are consistent with recent research demonstrating the uniqueness of the Tiwi population within the broader spectrum of Australian Indigenous populations. Silcocks et al. highlighted that 26% of the variants observed in Australian Indigenous cohorts were absent in individuals from Papua New Guinea or in the gnomAD dataset [10]. However, further admixture analyses are needed to confirm these findings. Constructing a cohort using a similar sequencing platform would enable more overlapping SNPs, accurate comparisons, and robust admixture estimates.

The ROH analysis in the Tiwi population revealed that this population has an elevated burden of ROH. Comparative analysis revealed that Tiwi individuals exhibited a higher frequency of ROH compared to several other populations, though their frequency remained lower than that of the Polynesian cohort. In contrast, the ROH frequency in the Melanesian population was lower than that observed in the Tiwi and Polynesian populations. The Pacific Islanders, including Polynesians, Melanesians, and Micronesians, underwent prolonged periods of isolation, which fostered genetic diversity between the islands [29]. This isolation remained largely uninterrupted until the arrival of the Western population during the 16^th^ and 19^th^ centuries [30], potentially contributing to the elevated burden of ROH observed in the Polynesian cohort compared to the Tiwi cohort. Furthermore, the Tiwi community exhibited a greater number of ROH compared to the American native populations, including the Amish and HUTT populations. These findings corroborate a recent study on Australian Aboriginal populations, which revealed that Tiwi genomes possess more than 10% extended homozygosity compared to other Indigenous communities. Moreover, functional variations are more likely to manifest in a homozygous state in Oceania compared to other regions [10].

The most prevalent ROH region was in chromosome 17 and corresponds to *FMNL1*, which encodes a formin-related protein involved in morphogenesis, cytokinesis, and cell polarity [31]. According to the Human Genetic Evidence (HuGE) [32] scores from the T2D database, *FMLN1* displays a robust association with weight (HuGE = 45.00), followed by a moderate association with diastolic blood pressure (9.03), and a moderate association with type 2 diabetes (7.75) [33]. Furthermore, the Kidney Tissue Atlas developed by the Kidney Precision Medicine Project (KPMP) revealed that *FMNL1* exhibits elevated RNA expression in acute kidney failure and CKD patients compared to healthy individuals [34]. These findings suggest that this genomic region may play a role in the pathogenesis of various diseases, especially kidney disease and type 2 diabetes. The second most prevalent ROH region was observed in chromosome 18 and includes part of the region covered by *ENSG00000286844* (uc288ffz). To the best of our knowledge, this region of the chromosome has not yet been completely characterized. Furthermore, we observed one of the longest ROH regions in chromosome 2 (2q32.3), which corresponds to *LINC01090*. The variants in this gene are associated with an increased risk of developing PTSD [35], and an increased risk of end-stage renal disease in type 2 diabetes [36]. These genomic regions may harbor functional variants that contribute to disorders such as PTSD and renal disease. However, most studies have focused on the European population, which highlights the need for further genetic research in Indigenous communities to confirm and validate these associations.

Here, we employed ROH mapping to identify genomic regions containing specific recessive genes and their genome-wide association with complex traits. We observed an association between elevated regional autozygosity and increased levels of urinary ACR at a specific region on chromosome 2 encompassing *FTO* and *CRNDE*. *FTO* is a protein-coding gene, and the variant within this gene is strongly associated with the pathogenesis of various diseases and disorders, including obesity, hypertension, and cardiovascular disorders such as heart failure, acute coronary syndrome, and aortic valve stenosis. Notably, being overweight represents a significant risk factor for CKD and is also associated with increased susceptibility to nephrolithiasis and several malignancies, including kidney cancer [37]. Another investigation revealed that *FTO* can modulate the m6A demethylation pathway to target mRNAs, influencing the onset and progression of cardiovascular diseases across different ethnic groups [38]. Our analysis of actual genotypes of variants in *FTO* based on their association with ACR levels revealed that five known independent variants exhibited a nominal association with ACR. Most importantly, the variant rs60286074 was associated with ACR (b = 0.0588, *p* = 0.00125) [39] in the Caucasian population. The independent variant rs55750853 was identified as a protective variant against kidney stone disease (Odds Ratio (OR) = 0.9142, *p* = 0.0025) [40]; however, in the Tiwi population, it was associated with increased ACR, reflecting the unique kidney profile of this population. Similarly, the variant rs118048822 was associated with an increased risk of obesity (OR = 1.25, *p* = 0.0018) in the Finnish (FinnGen) population [41]. These findings collectively suggest a significant association of *FTO* with kidney disease, hypertension, and cardiovascular disease. *CRNDE* is a long intergenic non-protein coding RNA gene that is transcribed into multiple transcript variants, some of which may function as non-coding RNAs [42]. The transcription of this gene is negatively regulated by insulin and insulin-like growth factors, and the gene product may regulate the expression of genes involved in metabolism [43]. The upstream variant found in this gene had a nominal association with increased ACR in the Tiwi population. Furthermore, *CRNDE* is also involved in biological pathways such as cell proliferation, differentiation, migration, and apoptosis, and it was found to be over-expressed in eight different types of cancers, including papillary renal cell carcinoma [44]; this led to speculation that it might be associated with kidney disease pathogenicity. In our validation using the recessive model, we identified a strong association between ACR levels and SNPs in the *FTO* and *CRNDE* gene regions. This finding reinforces our hypothesis that these regions are associated with kidney function. However, to our knowledge, we did not find any literature indicating that kidney function, particularly ACR, is predominantly recessive other than autosomal recessive polycystic kidney disease (ARPKD). This further highlights the unique genetic architecture of kidney function in the Tiwi Indigenous population.

Regional autozygosity association with ACR levels was observed in chromosome 19, encompassing members of the *PSG* family of genes, *CD177*, and *PRG1*. PSGs constitute a group of proteins important for pregnancy and fetal development. They are instrumental in modulating the maternal immune system’s reaction to the fetus, and are linked to immunomodulation and growth factor stimulation [45]. We also identified a nominal association between the SNPs in the *PSG1* gene and ACR levels under the recessive model. *CD177* is an important marker for neutrophil activation, demonstrating a strong correlation with serum levels, and it is further implicated in the severity and mortality of coronavirus disease. The evaluation of *CD177* as a dependable prognostic marker holds promise for routine clinical care for coronavirus patients [46]. The p53-responsive gene *PRG1* is an RNA gene predominantly associated with long non-coding RNA genes and may be involved in cellular processes regulated by p53 [31]. To the best of our knowledge, no prior studies have reported an association of this region with kidney disease/function.

Our study reveals the distinctive genetic profile of the Tiwi population, marked by a high prevalence of ROH and its association with clinical traits such as ACR. Significant links between genome-wide F_ROH_, regional autozygosity, and elevated ACR underscore the utility of these approaches in uncovering genetic factors influencing kidney function. Regional autozygosity mapping identified key associations on chromosome 2, including *FTO* and *CRNDE*, with variants in FTO associated with increased ACR levels, especially under the recessive model. These findings suggest potential screening tools for identifying individuals at risk of kidney disease and highlight the importance of further research into ROH and its role in disease pathogenesis. However, this study has certain limitations. The data for this study were generated using different platforms, including whole-genome sequencing and SNP genotyping arrays. To ensure comparability, we applied rigorous quality control and included only common SNPs identified across populations, minimizing potential platform-specific biases, and enhancing the consistency of the results. We identified only one trait associated with F_ROH_ and regional autozygosity, likely due to limited statistical power from the sample size and the low frequency of SNPs in ROH regions, which may have constrained the robustness of our estimates. Despite these limitations, the study provides a foundational framework for future research to explore the biological significance of ROH regions, the recessive nature of kidney function, and their impact on health within Indigenous populations.

## Supplementary information

Figure S1

Table S1 - S6

## Data Availability

The Tiwi data are stored in the QUT HPC Infrastructure in Brisbane. We have established a Data Access Advisory Committee (DAC), comprising Elders, Tiwi community members, and study investigators. Requests for external research access to Tiwi data will be evaluated by the DAC on a case-by-case basis, and access will be granted accordingly. This stringent review process is aligned with the strong recommendation from the Tiwi community and the Ethics Committee, which approved this research, to control and restrict access to Tiwi data. Requests to access the datasets should be directed to A/Prof Shiv Nagaraj. (shiv.nagaraj@qut.edu.au).

## Code availability

All original code has been deposited at the GitHub repository and is publicly available from https://github.com/va1208/Tiwi_ROH_analysis.
