# Supplementary material for: Regional autozygosity association with albumin-to-creatinine ratio reveals a novel FTO region in an Indigenous Australian population
Source: Eur J Hum Genet. 2025 Feb 24;33(12):1690–7. doi: 10.1038/s41431-025-01799-9 (PMC12669243; doi:10.1038/s41431-025-01799-9)
Supplement: Supplementary file 1 — Figure S1 [file 41431_2025_1799_MOESM1_ESM.docx]

**SUPPLEMENTARY FIGURES**


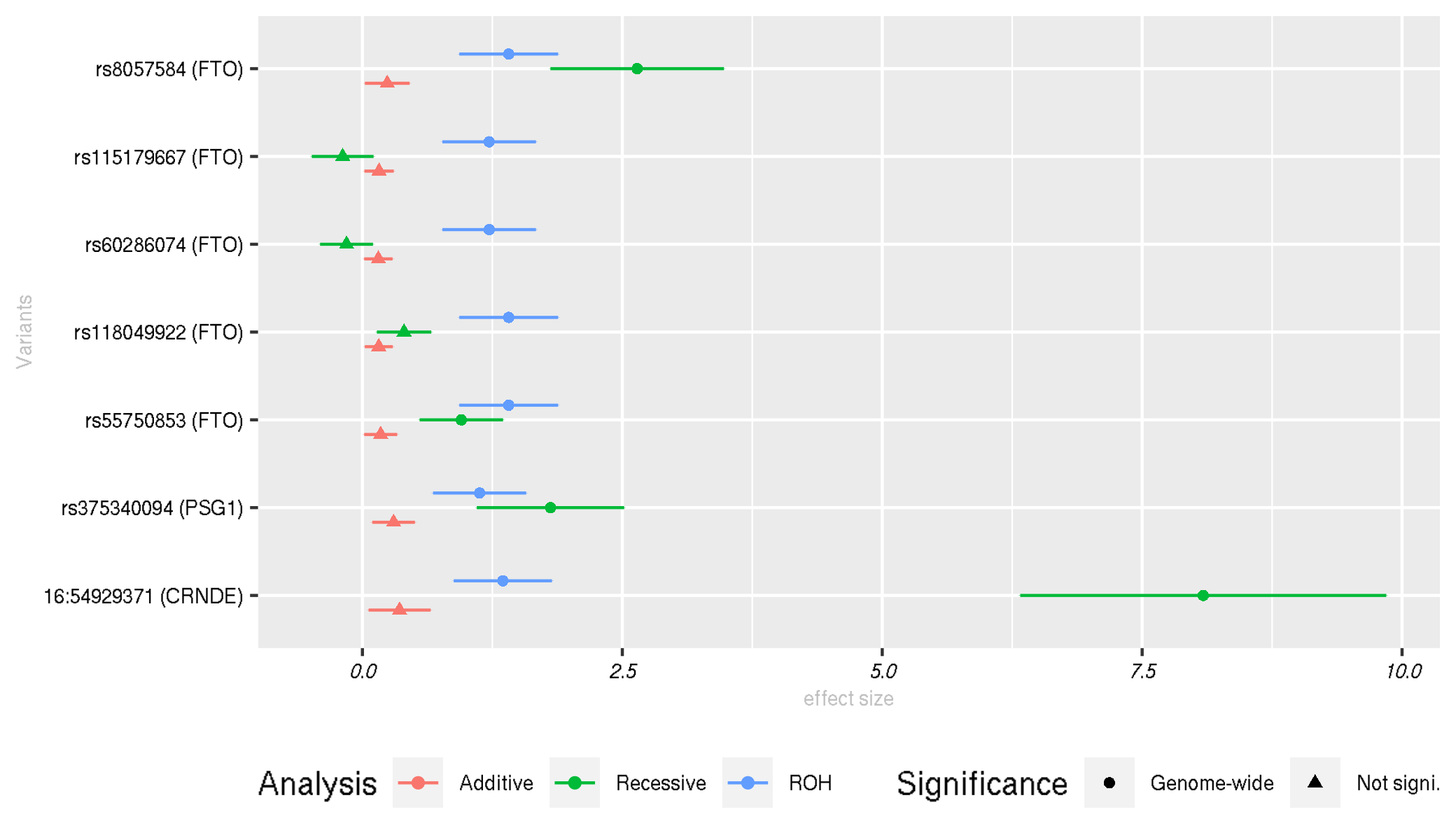


Figure S1: Effect sizes and 95% confidence intervals for the top independent regional autozygosity SNPs across three models: Additive GWAS, Recessive, and Regional Autozygosity. The shapes represent genome-wide significance, with dots indicating genome-wide significance and diamonds indicating SNPs that did not reach genome-wide significance. The corresponding gene annotations are provided in parentheses.
